# Theragnostic ^64^Cu/^67^Cu Radioisotopes Production With RFT-30 Cyclotron

**DOI:** 10.3389/fmed.2022.889640

**Published:** 2022-05-18

**Authors:** Jun Young Lee, Jung Ho Chae, Min Goo Hur, Seung Dae Yang, Young Bae Kong, Jongchul Lee, Jin Sik Ju, Pyeong Seok Choi, Jeong Hoon Park

**Affiliations:** Accelerator and Radioisotope Development Team, Korea Atomic Energy Research Institute, Daejeon, South Korea

**Keywords:** Copper-64, Copper-67, cyclotron, radioisotope, pair-radioisotope

## Abstract

^64^Cu and ^67^Cu are theragnostic pair radionuclides with promising application in the nuclear medicine. ^64^Cu is PET nuclide for the non-invasive diagnosis and ^67^Cu is beta emitter for therapy of various cancers. This study discusses optimization efforts in the production of these radioactive coppers carried out with 30 MeV cyclotron. Optimized conditions include target preparation, chemical separation, and quality control. The production routes of ^64^Cu and ^67^Cu were studied based on the nuclear reactions of ^64^Ni(p,n)^64^Cu and ^70^Zn(p,α)^67^Cu. The produced ^64^Cu and ^67^Cu have >99.9% of the radionuclidic purity. The yield at the end of bombardment (EOB) of ^64^Cu and ^67^Cu is 28.5 MBq/μAh and ^67^Cu is 0.58 MBq/μAh, respectively.

## Introduction

The nuclear medicine field relies on incorporating radioisotopes in small-molecule, nucleic acids, peptides, proteins, antibodies, and drug delivery technologies (1–3) that show high sensitivity for various diseases in order to impart diagnostic and therapeutic effects. Because of the same chemical properties, Copper-64 (^64^Cu) and (Copper-67) ^67^Cu can form chemical complexes using the identical labeling protocol, and diagnosis/therapy can be performed simultaneously (4–8). Copper is an essential trace element for the health of all living creatures. In humans, copper is necessary for proper function of organs and metabolic processes (9–13). Therefore, radioactive copper is a promising candidate that can be applied to various diseases. The multipurpose coordination chemistry of copper allows for its radiometallation with various chelators, such as DOTA (1,4,7,10-tetraazacyclododecane-tetraacetic acid), NOTA (1,4,7-triazacyclononane-triacetic acid), TETA (1,4,8,11-tetraazacyclotetraadecane-1,4,8,11-tetraacetic acid), and CB-TE2A (4,11-bis(carboxymethyl)-1,4,8,11-tetraazabicyclo[6.6.2] hexadecane) (14–17), which can be conjugated to various radiopharmaceuticals. ^64^Cu(T_1/2_: 12.7 h) is an attractive radioisotope of significant interest for positron emission tomography (PET) with β^+^(E_max_: 653.03 keV and E_mean_: 278.21 keV) and EC (electron capture: 1675.03 and 1345.77 keV) (18, 19). Furthermore, it has a relatively long half-life compared to fluorine-18 (T_1/2_: 110 min) and carbon-11 (T_1/2_: 20.4 min) (19, 20), which corresponds to an adequate half-life for drug requiring long-term follow-up. ^67^Cu(T_1/2_ = 61.83 h, β^−^ mean energy = 141 keV) is a radioisotope with significant potential for therapeutic applications in nuclear medicine due to a similar beta mean energy as 134 keV of ^177^Lu (21–24, 26). Despite its potential, the use of ^67^Cu for radionuclide therapy has been hindered for decades by its limited supply and low-specific activity. However, the production of ^67^Cu has been attempted through various nuclear reactions, such as ^68^Zn(p,2p)^67^Cu, ^70^Zn(p,α)^67^Cu, ^67^Zn(n,p)^67^Cu, and ^68^Zn(γ,p)^67^Cu (25– 31). In this study, the irradiation, target preparation, chemical separation, and quality control of radioactive copper (^64^Cu and ^67^Cu) were verified. The results show that optimized enriched target electrodeposition, proton beam irradiation, separation/purification, and quality control processes can enhance the routine capability of ^64^Cu and ^67^Cu production.

## Materials and Methods

**Figure 1:**
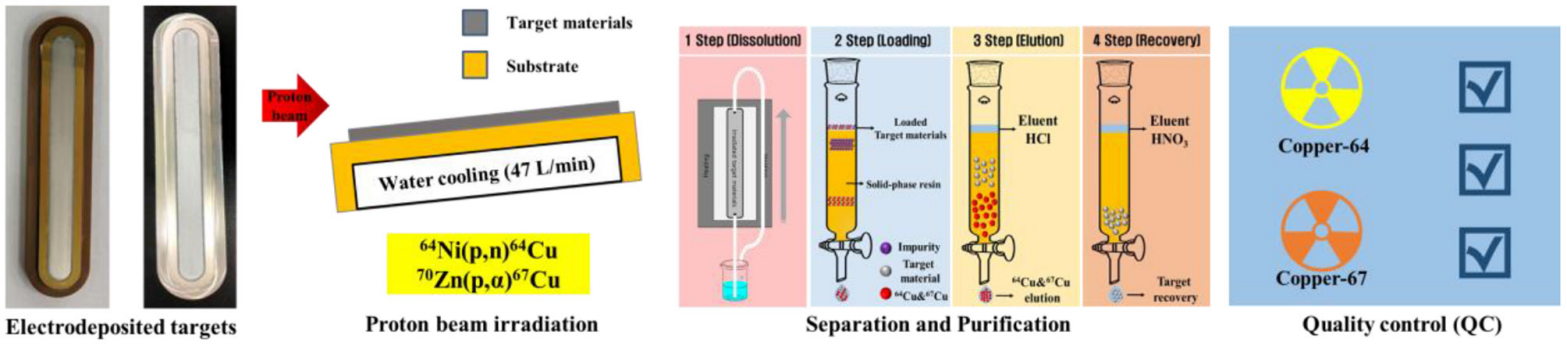
Schematic illustration of pair-radioisotope production route.

As shown in Figure 1 below, radioisotopes are produced with a proton beam irradiation process to ^64^Ni and ^70^Zn electrodeposited target. In order to maximize the target cooling and the production efficiency of ^64^Cu and ^67^Cu when irradiating the target material with a proton beam, a tilted target and a backside water cooling system were applied. The radioactive copper from the target materials was carried out through a solid-phase separation method. The steps of impurities removal, radioactive copper purification, and target material recovery were performed. Finally, ^64^Cu and ^67^Cu were verified through quality control of radionuclidic purity and impurity metal content.

### Materials and Reagents

Ultra-high purity reagents were used for production of ^64^Cu and ^67^Cu in this study. Isotopically enriched ^64^Ni (^58^Ni: 0.05%, ^60^Ni: 0.03%, ^61^Ni: 0.004%, ^62^Ni: 0.396%, ^64^Ni: 99.52%) and ^70^Zn (^64^Zn: 0.1%, ^66^Zn: 0.1%, ^67^Zn: 0.1%, ^68^Zn: 2.2%, ^70^Zn: 97.5%) were supplied from ISOFLEX (San Francisco, CA, USA). The proton beam verification film (Gafchromic Quick Phantom) was obtained from Ashland Company (Gafchromic^TM^ Ashland Inc., New Jersey, USA). Substrates of Au-Cu and Ag were received from Doowon Machinery Company (Seoul, South Korea) in a bar shape (1 x 12 cm). Concentrated HCl was purchased from Thermo Fisher Scientific (Waltham, MA, USA); Hydrazine hydrate and sodium hydroxide were purchased from Sigma-Aldrich (St. Louis, MO, USA); CU resin and ZR cartridge were obtained from TRISKEM Company (Bruz, Brittany, France); and AG1X8 anion exchange resin was obtained from Bio-Rad Laboratories (Hercules, CA, USA).

### Equipment

The proton beam irradiation studies were performed using a RFT-30 cyclotron (30 MeV, Korea Atomic Energy Research Institute). The apparatus for enriched target material electroplating and dissolution was developed in house. For ^64^Cu and ^67^Cu radioactivity measurement, an ionization chamber (AtomlabTM500, BIODEX, New York, USA) was used. The radionuclidic purity was measured by a calibrated high purity germanium gamma detector (HPGe, Oak Ridge, USA). An inductively coupled plasma-mass spectrometry (ICP-MS) system (Agilent 7500, Stevens Creek Blvd, CA, USA) was used to analyze the metallic impurity of the final eluted radioactive copper solution.

### Target Preparation

The electroplated target was prepared by an electrodeposition procedure. A typical electroplating target material (^64^Ni or ^70^Zn) was dissolved in 10 mL of concentrated HCl. After the target metal was completely dissolved, the target solution was then evaporated to dryness under a vacuum system. The residue was re-dissolved in 600 mL of water, and then 2 mL of hydrazine hydrate was added as an electrolyte to the target solution. The final solution was loaded into the electroplating cell with the substrate. The electroplating was carried out on the substrate at optimized conditions (waveform: square, frequency: 50 Hz, amplitude: 2000, tau: 2, phase: 10 deg, chopping frequency and duty: 100 Hz and 84%, square duty: 60%). After electroplating, the ^64^Ni and ^70^Zn targets were examined by measuring their thickness and uniformity.

### Proton Beam Irradiation

^64^Cu and ^67^Cu were produced at RFT-30 MeV cyclotron by ^64^Ni(p,n)^64^Cu and ^70^Zn(p,α)^67^Cu nuclear reaction, respectively. The electroplated target was fixed on the solid target station and irradiated with 11.0 MeV (^64^Cu) and 17.7 MeV (^67^Cu) protons. The solid target station was fitted with a self-made cradle for the tilted 6° target compared to the beam line. Using Gafchromic film, we optimized the target area of 1175 mm^2^ with a beam distribution of 90% under the same conditions as employed for ^64^Cu and ^67^Cu. Furthermore, the beam currents and cooling system were considered for reducing target thermal damage. The central cooling-water system was connected to the target station (water-pressure: 1.1 MPa, water cooling line: 1/4”). For the production of ^64^Cu and ^67^Cu, the beam current was fixed at 30 and 100 μA for 3 and 12 h, respectively.

### Separation and Purification

#### Copper-64

The irradiated ^64^Ni target (Electroplated target weight: 130 mg) was directly transported to the hot-cell using an automatic target transport system. The irradiated target was dissolved in 7 mL of 8 M HCl with a target dissolving device at 90°C for 1.5 h. The recovered target solution was filtered with a 0.45 μm PVDF syringe filter. To adjust the pH of the dissolved target solution to 2, the solution was evaporated and re-dissolved with water. ^64^Cu was separated using copper selective CU resin. In brief, 300 mg of CU resin was immersed in water to remove air bubbles and then left in a vacuum for 30 min at 8 mbar. In the wet-packing method, the empty column was filled with immersed CU resin and then the solution was replaced with 0.01 M HCl for the pre-conditioning column. The proton beam irradiated crude ^64^Ni target solution was loaded on the CU resin pre-packed column at a concentration of 0.01 M HCl. After loading the ^64^Cu and other radio impurities, the CU resin was washed with 20 mL of 0.01 M HCl (1.0 mL/min) to recover and remove the ^64^Ni target and impurities. Finally, the ^64^Cu was eluted with 2 mL of 8 M HCl, and then ^64^Cu fractions were collected and then evaporated nearly to dryness under a vacuum system.

#### Copper-67

The separation and purification of the carrier-free ^67^Cu were performed as follows. After irradiation, the ^70^Zn target (electroplated target weight: 260 mg) was placed into the target dissolving device. The ^70^Zn target was dissolved in 7 mL of 9 M HCl at 90°C for 10 min. The dissolved target solution was filtered with a 0.45 μm PVDF syringe filter. We used a two-step separation procedure to remove ^66^Ga impurities and recover the ^70^Zn target materials with a ZR cartridge and AG1X8 ion exchange resin. In brief, the target solution was loaded into a ZR cartridge to remove ^66^Ga and then the cartridge was washed with 9 M HCl for complete recovery of ^70^Zn and ^67^Cu. The eluted solution was passed through a wet-packed AG1X8 column (packing height: 7 cm). The column was then washed with 2.5 column volumes of 9 M HCl to eliminate other impurities. The ^67^Cu was eluted with 8 mL of 2 M HCl, and then ^67^Cu fractions were collected and then evaporated nearly to dryness under a vacuum system. Finally, enriched ^70^Zn was completely recovered with 30 mL of 2 M HNO_3_ and then evaporated for future use.

## Quality Control

### Metallic Impurity

The impurity metal content (V, Cr, Mn, Fe, Co, Ni, and Zn) of the separated and purified radioactive copper (^64^Cu and ^67^Cu) was evaluated via an inductively coupled plasma-mass spectrometer (ICP-MS) to secure biological safety and optimize the radio-labeling yield. The operating conditions were as follows: instrument (Agilent 7500 series), nebulizer (Babington type), spray-chamber (Scott-type), FR generator (frequency: 10 MHz, power 1,300 W), Ar flow rate (plasma 15 L/min, auxiliary 0.9 L/min, nebulizer 1 L/min), sample uptake rate 1 mL/min, and number of replicates (three).

### Radionuclidic Purity

Radionuclidic purity was determined using gamma spectroscopy with a high purity germanium detector, multichannel analyzer, and Gamma Vision software. Efficiency and energy calibration was performed with ^210^Pb (401 Bq/g; 57 keV), ^241^Am (40 Bq/g; 60 keV), ^109^Cd (385 Bq/g; 88 keV), ^57^Co (15 Bq/g; 122 keV), ^123m^Te (22 Bq/g; 159 keV), ^51^Cr (491 Bq/g; 320 keV), ^113^Sn (73 Bq/g; 392 keV), ^85^Sr (92 Bq/g; 514 keV), ^137^Cs (65 Bq/g; 662 keV), ^88^Y (149 Bq/g; 898 and 1,836 keV), and ^60^Co (77 Bq/g; 1,173 and 1,333 keV). Activity of the multi-nuclide standard source was checked at the day of measurement. The purified samples were fixed on a universal sample holder located 5 cm from the detector window. The gamma spectra were recorded for 86,400 s each for the crude target solution and purified product.

## Results

### Target Manufacturing

**Figure 2:**
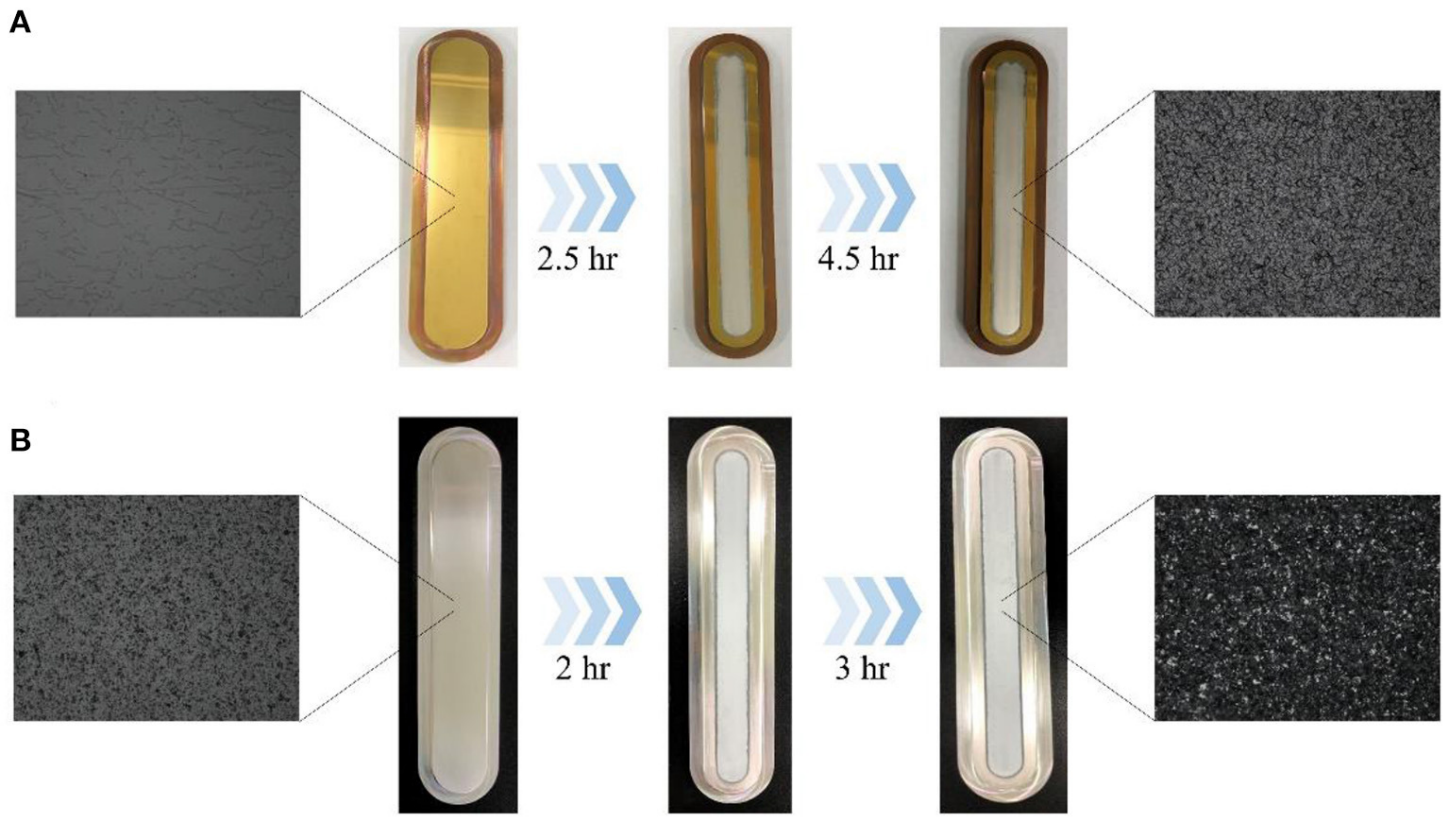
Enriched target electroplated onto substrate (Au-Cu and Ag) **(A)** ^64^Ni electrodeposition with Au-Cu substrate **(B)** ^70^Zn electrodeposition with silver substrate (magnification under optical microscope X20).

To optimize the production of ^64^Cu and ^67^Cu via the nuclear reaction of ^64^Ni(p,n)^64^Cu and ^70^Zn(p,α)^67^Cu, we prepared ^64^Ni and ^70^Zn targets on the substrate by electrodeposition. Gold and silver were used as a cathode and a platinum rod was used as an anode. The bar-shaped substrates were electrodeposited with ^64^Ni and ^70^Zn. After cleaning and drying, the weight of the electroplated ^64^Ni and ^70^Zn on the substrate was 130 and 260 mg, respectively. The target having a uniform surface was confirmed through an optical microscope (Figure 2; Supplementary Figure 4).

### Proton Beam Irradiation

Enriched ^64^Ni and ^70^Zn targets were mounted using a self-produced cradle (6° tilted target system) and a target transfer device and then a proton beam was irradiated at 11 and 17.7 MeV incident energy, respectively. We predicted the nuclear reaction cross-section for ^64^Cu and ^67^Cu production as 800 and 15 mb at 11 and 17.7 MeV incident energies, respectively, through the NNDC (National nuclear data center) database. The nuclear cross-sections of ^64^Cu and ^67^Cu are detailed in the Supplementary Figures 1–5. Based on the theoretical calculation results, the proton beam was irradiated with accumulated current of 90 and 1,200 μAh. The purified ^64^Cu and ^67^Cu were produced and isolated from irradiated ^64^Ni and ^70^Zn targets; considering the correction due to the decay, at the end of bombardment (EOB) we obtained ^64^Cu 28.5 MBq/μAh and ^67^Cu 0.58 MBq/μAh.

### Separation and Purification

#### Preparation of ^64^Cu

**Figure 3:**
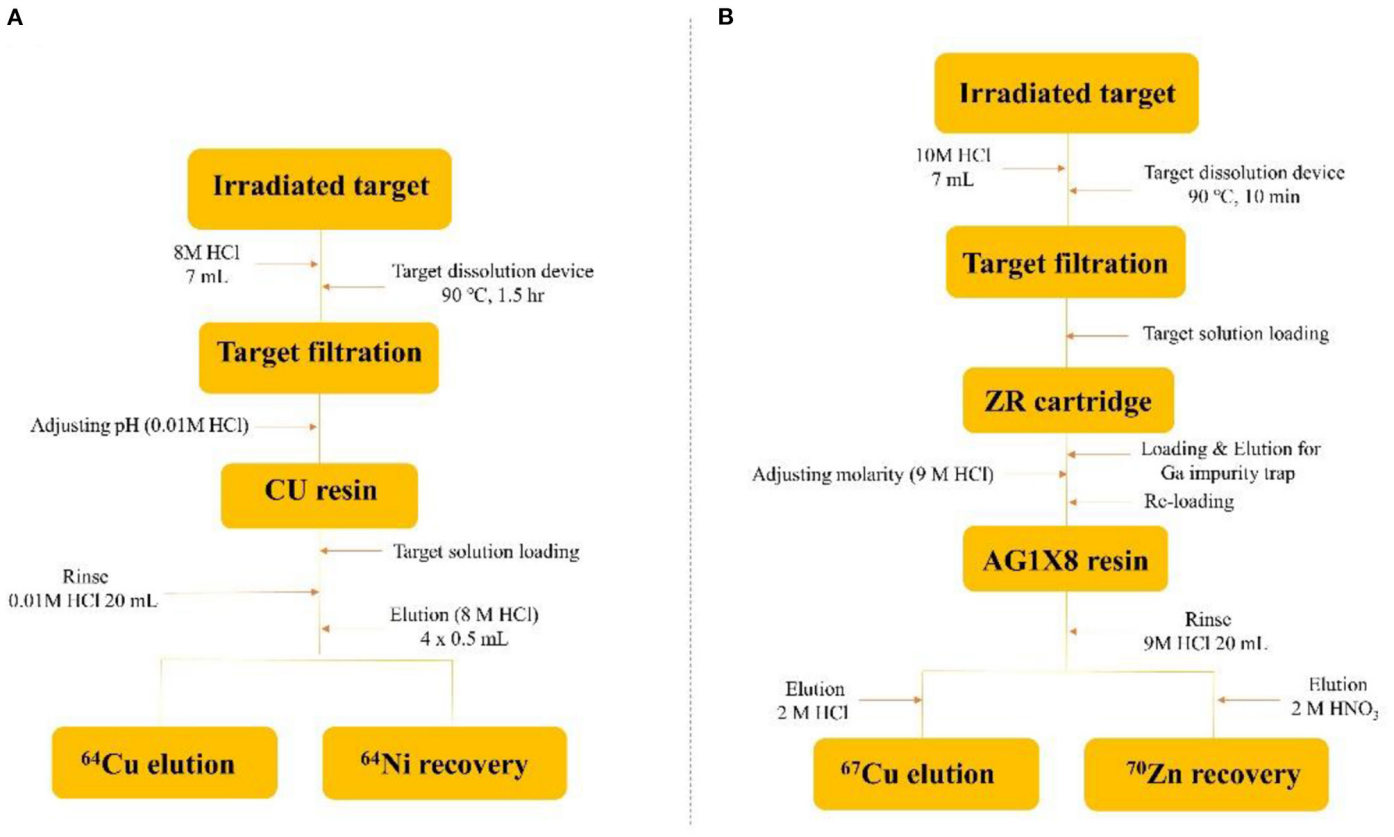
**(A,B)** Schematic of chemical separation processing.

**Figure 4:**
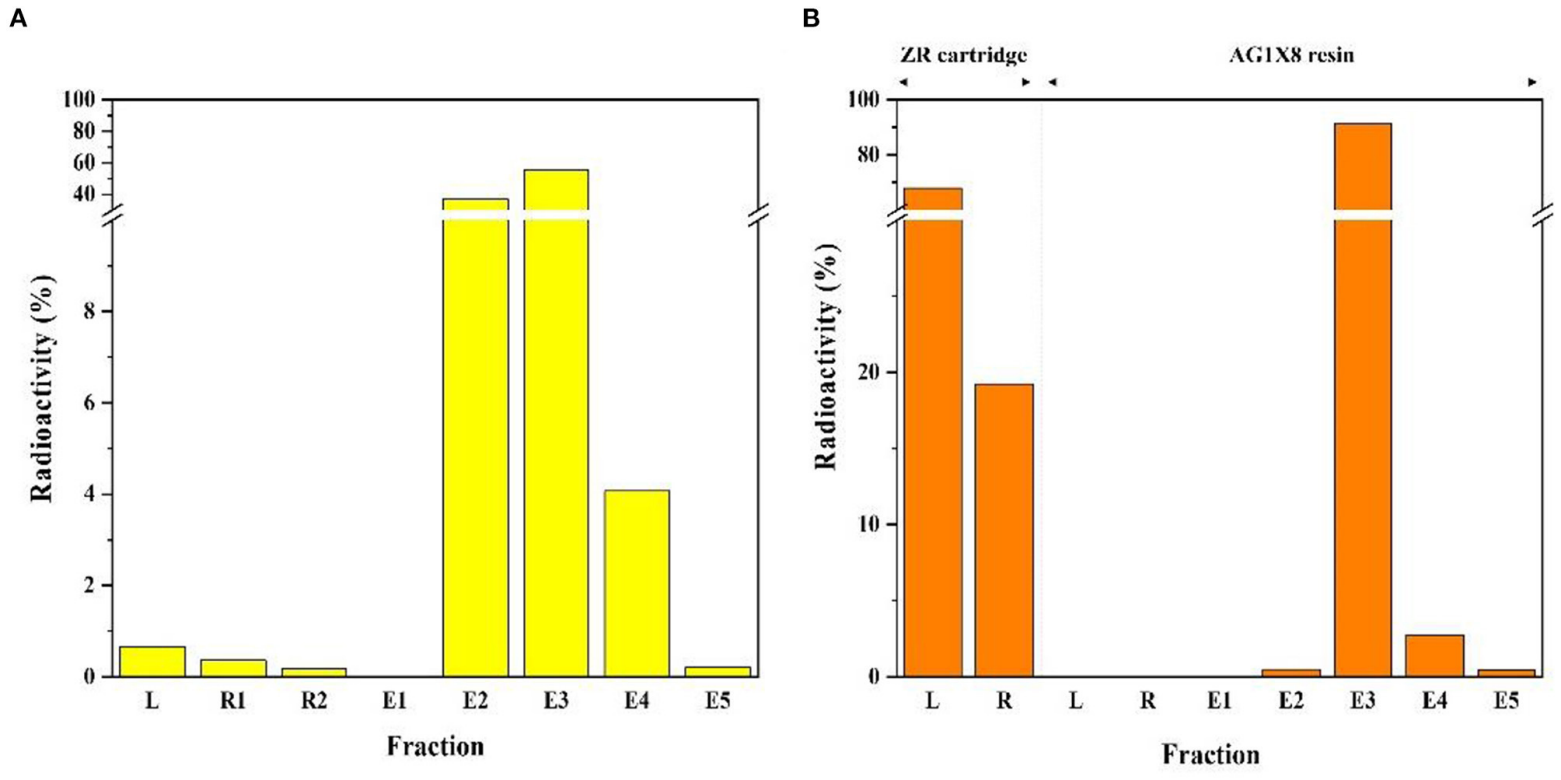
Elution profile of radioactive copper separation from the irradiated target materials **(A)** ^64^Cu and **(B)** ^67^Cu.

After irradiation for three hours, the ^64^Cu was completely separated from the enriched ^64^Ni target solution (Figure 3A). Figure 4A shows the elution profile obtained from the measurement of the fraction activity from the CU resin. To elute ^64^Cu from CU resin, ^64^Cu was eluted four times with a volume of 500 μL at a flow rate of 0.5 mL/min using 8 M HCl (^64^Cu elution yield: 98.6%). The radioactivity of purified ^64^Cu in each 500 μL fraction and the residual activity on the column were measured by using a dose calibrator. To improve the availability of ^64^Cu in mild labeling conditions, the purified ^64^Cu solution was evaporated and re-dissolved in 0.1 M HCl. Finally, ^64^Ni was collected during the separation and purification procedure for the target recycling.

#### Preparation of ^67^Cu

After irradiation for 12 h, the enriched ^70^Zn target was completely dissolved in 9 M HCl. The target solution was chemically processed using two-step separation with a solid-phase resin, specifically, a ZR cartridge and AG1X8 resin (Figure 3B). The target solution was passed through the ZR cartridge to remove ^66^Ga radio metallic impurities. The ^66^Ga was completely removed from the crude target solution and then fractions were collected for subsequent separation. During ^67^Cu production, ~2 days of cooling time was required to remove ^66^Ga. To maximize the production yield, the method of removing ^66^Ga was adopted within 20 minutes. Figure 4B shows the elution profile obtained from the measurement of fraction activity from the collected solution. AG1-X8 ion exchange resin was c in water and transferred into an empty column (1 x 10 cm). The target solution was passed through pre-conditioned AG1X8 resin. To elute ^67^Cu and ^70^Zn from the AG1X8 resin, 2 M HCl and 2 M HNO_3_ were used at a 1.0 mL/min flow rate, respectively.

## Quality Control

**Figure 5:**
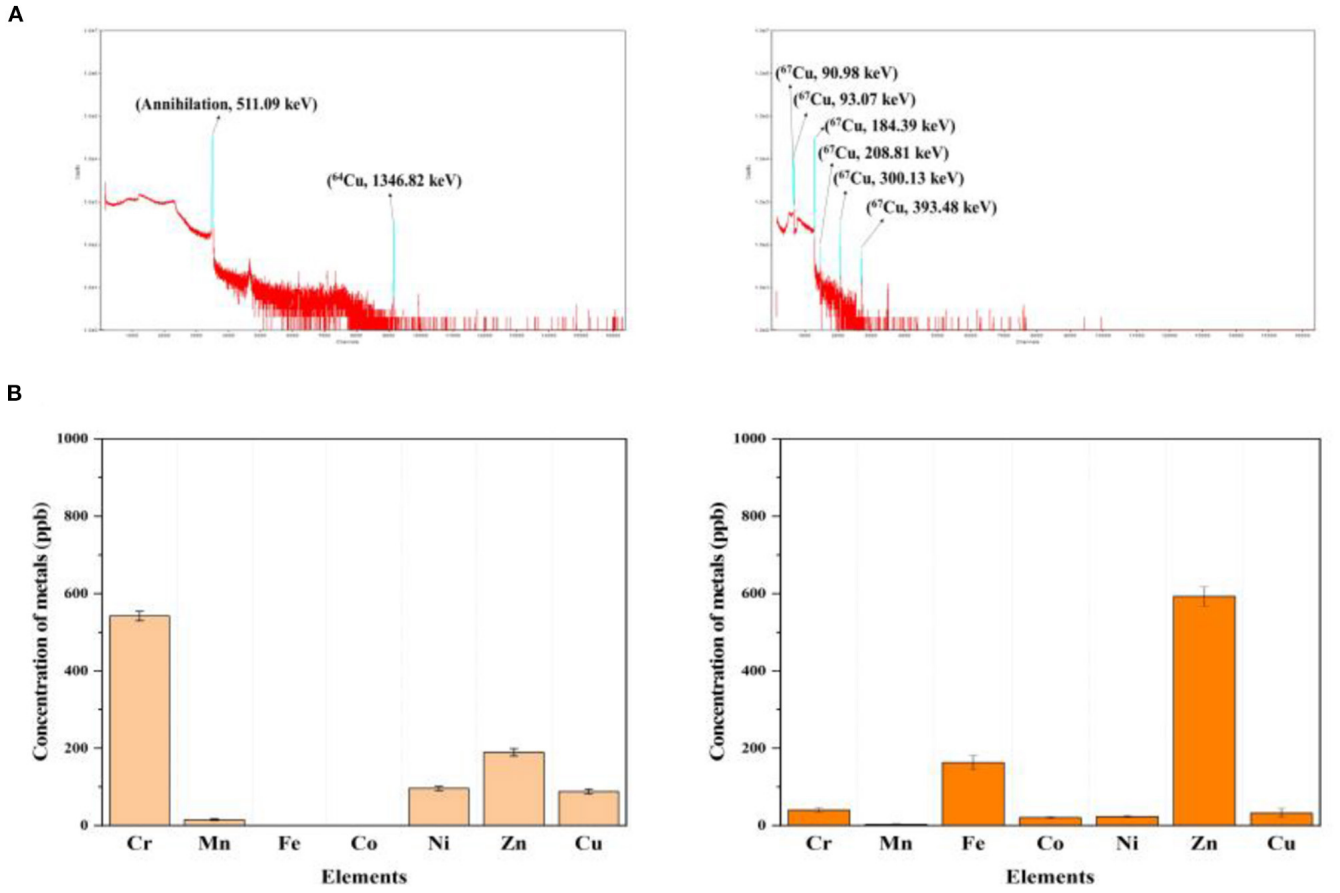
Quality control of pair-radioisotopes **(A)** gamma energy spectrum of purified ^64^Cu (left) and ^67^Cu (right), **(B)** metallic impurity content in purified final volume of ^64^Cu (left) and ^67^Cu (right).

The radionuclidic purity of ^64^Cu and ^67^Cu in the separated fraction volume was assessed using a high purity germanium detector. The radioisotope production routes involved radionuclidic impurities that should be removed by chemical separation before use. The gamma energy of the final product must be measured. Figure 5A shows >99% high purity γ-ray spectra of purified ^64^Cu and ^67^Cu. In addition, gamma energy spectra of proton beam irradiated target materials described in the Supplementary Figures 1–5. Metallic impurities of the ^64^Cu and ^67^Cu elute were determined using ICP-MS to identify metal species that may compete with copper during radio-labeling reactions for radiopharmaceuticals. The content of metallic impurities in the purified ^64^Cu and ^67^Cu is <1 ppm, as shown in Figure 5B, and it will not affect the radio-labeling reaction (Supplementary Figure 5).

## Discussion and Conclusions

The production procedure of pair-radioisotope ^64^Cu and ^67^Cu has been studied. This attempt is an imperative study to guarantee the supply and quality of radioisotopes for diagnosis/therapy in the field of nuclear medicine. Each condition was established using RFT-30 cyclotron, equipment, and chemicals to optimize the entire process, including proton beam irradiation, targetry, chemical separation, and quality control of ^64^Cu and ^67^Cu. The expected thick target yields of ^64^Cu and ^67^Cu were calculated considering the NNDC cross-sections with 11 and 17.7 MeV as incident energy, respectively. The obtained experimental production yields were 2.57 GBq and 696 MBq, respectively. These results have secured sufficiently radioactivity that can be supplied to researchers in the field of nuclear medicine. Furthermore, we will continue to conduct research for mass production. Preparation of electrodeposited target has several advantages including high solidity, density, and heat dissipation efficiency for beam irradiation. Moreover, it is helpful for tilted targets to secure the maximum beam irradiation area. Chemical separation studies were performed using ZR cartridge and AG1X8 resin for ^67^Cu and CU resin for ^64^Cu, respectively, considering the copper adsorption capacity, target material recovery, and impurity removal. ^64^Cu and ^67^Cu were prepared with >98% separation efficiency, and the final product contains the maximum radioactivity in the minimum volume to introduce the radioactive copper in the radiopharmaceutical under mild reaction conditions, it was evaporated and dried and then re-dissolved in 0.1 M HCl. Furthermore, the radiopharmaceuticals limit the amount of radionuclide contamination allowed in ^64^Cu and ^67^Cu solutions for safety and radio-labeling efficiency. The major radionuclide contaminant of radioactive copper solutions is its target material, such as nickel or zinc. Through the quality control procedure, the radionuclidic purity of the final eluted solution was measured as >99% and the metallic impurity content was maintained less than 1 ppm. Further studies on the automation separating apparatus are underway for ^64^Cu and ^67^Cu production.

## Data Availability Statement

The original contributions presented in the study are included in the article/Supplementary Material, further inquiries can be directed to the corresponding author.

## Author Contributions

JYL and JC: conceptualization, data curation, formal analysis, methodology, visualization, writing—original draft, and investigation. JP: funding acquisition and project administration. MH, SY, YK, JL, and JJ: cyclotron operating. JP and SY: validation and writing—review and editing. All authors have read and agreed to the published version of the manuscript.

## Funding

This research was supported by the Nuclear R&D Program through the National Research Foundation of Korea, funded by the Ministry of Science, ICT, and Future Planning (2017M2A2A6A05016600 and 2021M2E7A1079112) and NTIS (1711123216), Republic of Korea.

## Conflict of Interest

The authors declare that the research was conducted in the absence of any commercial or financial relationships that could be construed as a potential conflict of interest.

## Publisher's Note

All claims expressed in this article are solely those of the authors and do not necessarily represent those of their affiliated organizations, or those of the publisher, the editors and the reviewers. Any product that may be evaluated in this article, or claim that may be made by its manufacturer, is not guaranteed or endorsed by the publisher.
