# Supplementary material for: Theragnostic 64Cu/67Cu Radioisotopes Production With RFT-30 Cyclotron
Source: Front Med (Lausanne). 2022 May 18;9:889640. doi: 10.3389/fmed.2022.889640 (PMC9158440; doi:10.3389/fmed.2022.889640)
Supplement: Supplementary file 1 [file Data_Sheet_1.docx]

Supplementary Material


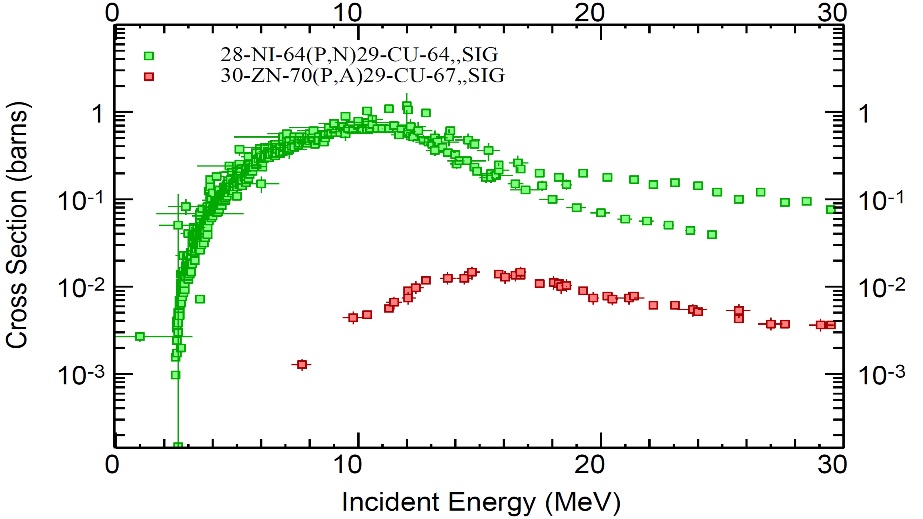


**Supplementary Figure 1.** Cross-sections of proton induced reactions on ^64^Ni and ^70^Zn: production of ^64^Cu and ^67^Cu.


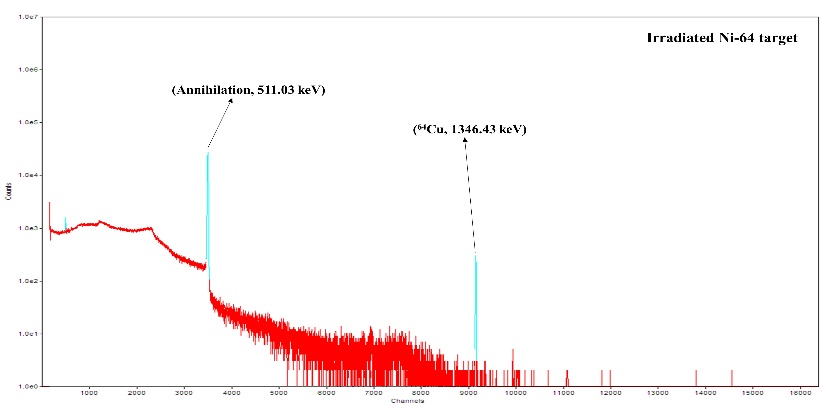


**Supplementary Figure 2.** Gamma spectrum of the irradiated ^64^Ni target solution.

**
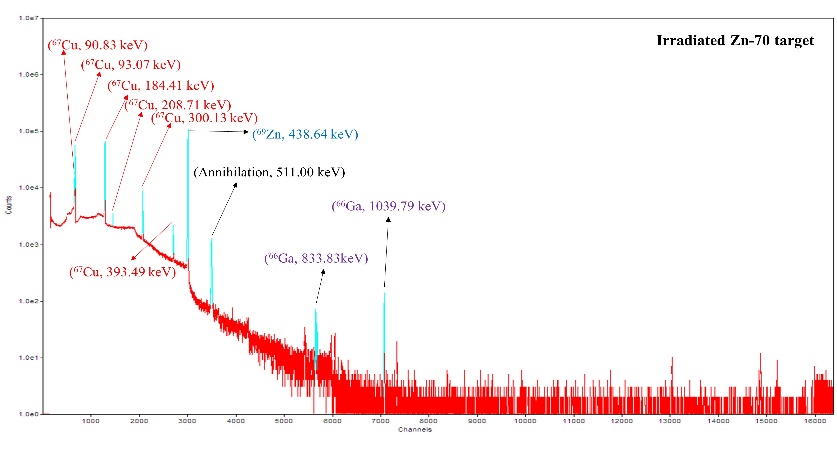
**

**Supplementary Figure 3.** Gamma spectrum of the irradiated ^70^Zn target solution.

**
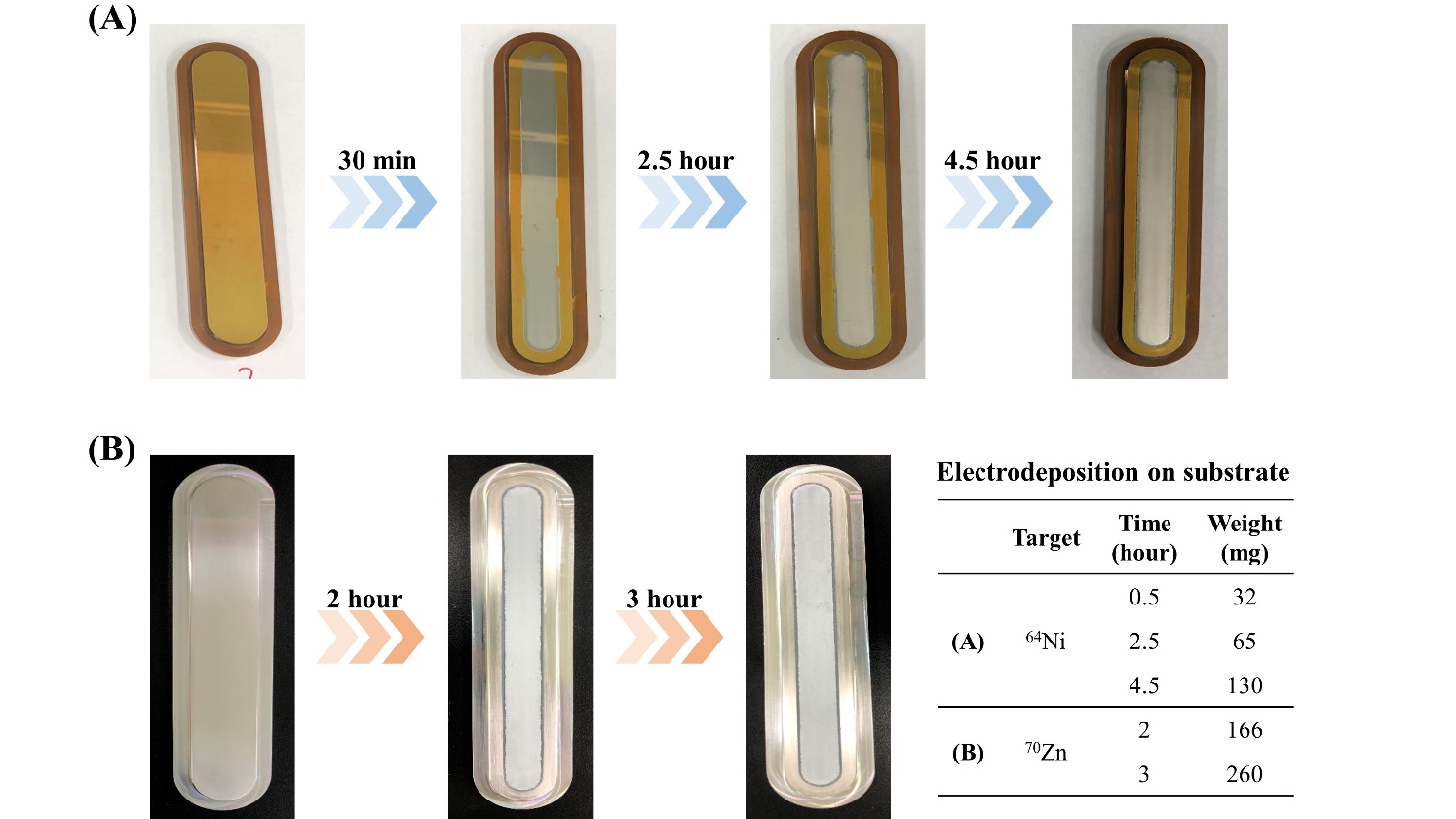
**

**Supplementary Figure 4.** Target preparation of (A) ^64^Ni and (B) ^70^Zn via electrodeposition method.

**
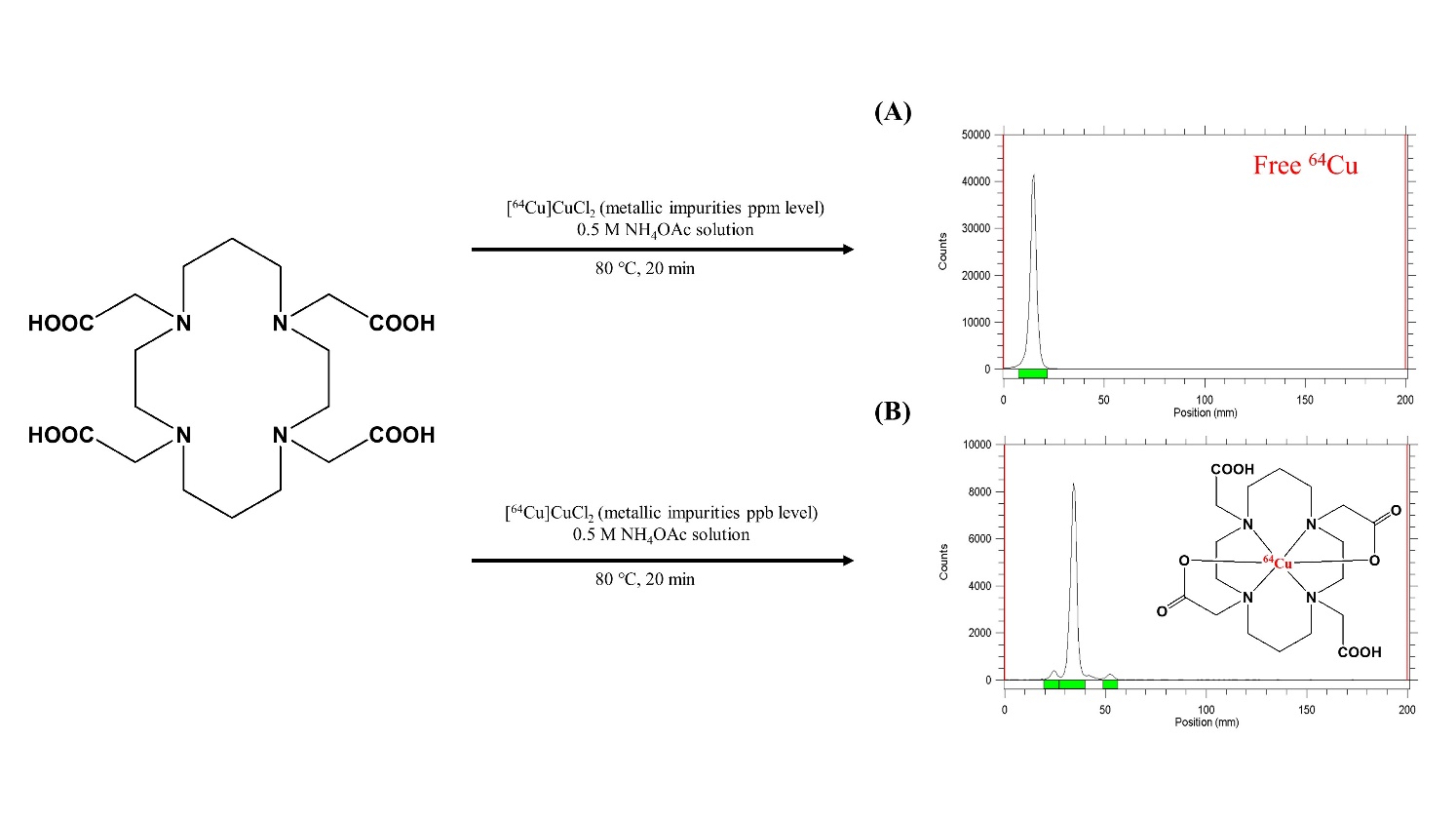
**

**Supplementary Figure 5.** ^64^Cu chelation chemistry with TETA (A) ^64^CuCl_2_ containing metal impurities with ppm level (B) ^64^CuCl_2_ containing metal impurities with ppb level.
